# A Streamlined Method for Detecting Structural Variants in Cancer Genomes by Short Read Paired-End Sequencing

**DOI:** 10.1371/journal.pone.0048314

**Published:** 2012-10-29

**Authors:** Martina Mijušković, Stuart M. Brown, Zuojian Tang, Cory R. Lindsay, Efstratios Efstathiadis, Ludovic Deriano, David B. Roth

**Affiliations:** 1 Department of Pathology and Laboratory Medicine and Abramson Family Cancer Research Institute, Raymond and Ruth Perelman School of Medicine, University of Pennsylvania, Philadelphia, Pennsylvania, United States of America; 2 Center for Health Informatics and Bioinformatics, New York University Langone Medical Center, New York, New York, United States of America; 3 Department of Immunology, Institut Pasteur, Paris, France; 4 Centre National de la Recherche Scientifique URA1961, Paris, France; Duke-National University of Singapore Graduate Medical School, Singapore

## Abstract

Defining the architecture of a specific cancer genome, including its structural variants, is essential for understanding tumor biology, mechanisms of oncogenesis, and for designing effective personalized therapies. Short read paired-end sequencing is currently the most sensitive method for detecting somatic mutations that arise during tumor development. However, mapping structural variants using this method leads to a large number of false positive calls, mostly due to the repetitive nature of the genome and the difficulty of assigning correct mapping positions to short reads. This study describes a method to efficiently identify large tumor-specific deletions, inversions, duplications and translocations from low coverage data using SVDetect or BreakDancer software and a set of novel filtering procedures designed to reduce false positive calls. Applying our method to a spontaneous T cell lymphoma arising in a core RAG2/p53-deficient mouse, we identified 40 validated tumor-specific structural rearrangements supported by as few as 2 independent read pairs.

## Introduction

Somatic structural variants (SVs), including large deletions, insertions, inversions, duplications and translocations are important hallmarks of cancer genomes, responsible for the creation of fusion genes, copy number and regulatory changes leading to activation or overexpression of oncogenes and inactivation of tumor suppressor genes [1], [2], [3], [4], [5], [6]. Defining the architecture of a specific cancer genome is therefore essential not only as a first step towards understanding the biology of the tumor and mechanisms of oncogenesis, but also clinically towards designing effective personalized therapies [7], [8].

Recent advances in high throughput sequencing technology [9], [10] have made it possible to study whole genomes at unprecedented high resolution and relatively low cost. However, the current short read paired-end sequencing technologies carry many challenges, especially apparent when attempting to study SVs in cancer. First, the inherent complexity of tumor tissue [11], [12], [13] is a challenge in itself, since tumors are rarely monoclonal and are often mixed with normal tissue, so the sequencing coverage must be deeper than for SV detection in the germline. Second, short reads generated by paired-end sequencing (typically, 50–100 bp from each end of the 300–400 bp DNA fragment) prove to be difficult to map correctly back onto the reference genome due to the high percentage of repetitive genomic sequences [14], [15], [16], [17]. All this leads to a large number of false positive calls, generating unacceptable levels of noise. Retrotransposon activity, common in human and mouse genomes [18], [19], additionally complicates the data analysis leading to certain types of false positive calls. Finally, DNA library preparation artefacts arising from PCR amplification combined with sequencing errors add another level of complexity.

This work describes a whole genome sequencing based approach to identify 4 types of SVs: large deletions, inversions, duplications and translocations. We used SVDetect [20] and BreakDancer [21] to call SVs in a mouse lymphoma genome from a set of paired-end reads obtained on the Illumina’s HiSeq platform. In order to reduce the high number of false positive calls, we developed a filtering procedure that allows detection of tumor-specific events at relatively low coverage (17x). First, we found it essential to compare the tumor dataset to a germline sample obtained from the same animal, to remove a large number of germline SVs (mostly arising from retrotransposon activity) detected in the experimental animal when compared to the reference genome. Second, we developed methods to remove read pairs marked as discordant due to alignment errors, as well as imperfect PCR duplicates arising from DNA library preparation and sequencing errors. Third, we applied several filters on the results produced by SV calling programs, such as overlaps with annotated simple repeats and low mappability regions, in order to identify high confidence SV candidates. We show PCR and Sanger sequencing validation of 40 tumor-specific SVs in a single tumor genome supported by as few as 2 independent read pairs.

In summary, the method presented here simplifies the analysis, increasing sample throughput. It also provides high sensitivity, allowing detection of rare variant clones in complex mixtures that may have important prognostic or therapeutic consequences.

## Results and Discussion

### Establishing Initial Analysis Parameters

We used paired-end (PE) sequencing simulations as a tool to establish the initial analysis parameters, to quantify the effect of sequencing depth on detection of known SVs, and to study alignment related false positives. We simulated a rearranged genome based on C57BL/6J mouse reference (mm9), introducing 10 interchromosomal translocations and 10 large deletions into areas of varying mappability (Table 1). Read length, mean insert size and standard deviation of the insert size were chosen to be representative of our experimental data (50, 315, 44, respectively). Using three independent simulated datasets with 10, 20, 40, 80 and 160 million read pairs, we assessed the number of detected real and false positives, as well as the detection probability as a function of local mappability.

**Table 1:** List of simulated SVs with mappabilities.

SV*	Size (bp)	Mappability (%)
TR 15_12	–	100
TR 12_15	–	100
TR 10_X	–	50
TR X_10	–	50
TR 16_6	–	70.2
TR 6_16	–	52.8
TR 7_11	–	66
TR 11_7	–	73.8
TR 14_13	–	18.5
TR 13_14	–	36.4
DEL 1	576,373	100
DEL 2	46,610	95.1
DEL 3	600,033	85.3
DEL 4	5,963	100
DEL 5	64,735	100
DEL 8	1,433	77.4
DEL 9	10,789	100
DEL 17	3,066	100
DEL 18	1,000,440	100
DEL 19	21,449	100

* TR = translocation, DEL = deletion. Numbers show chromosome(s) involved.

Mappability is calculated as percentage of 50 bp windows with 100% mappability obtained from the UCSC Table Browser inside the region of 265 bp on each side of the breakpoint.

**Figure 1:**
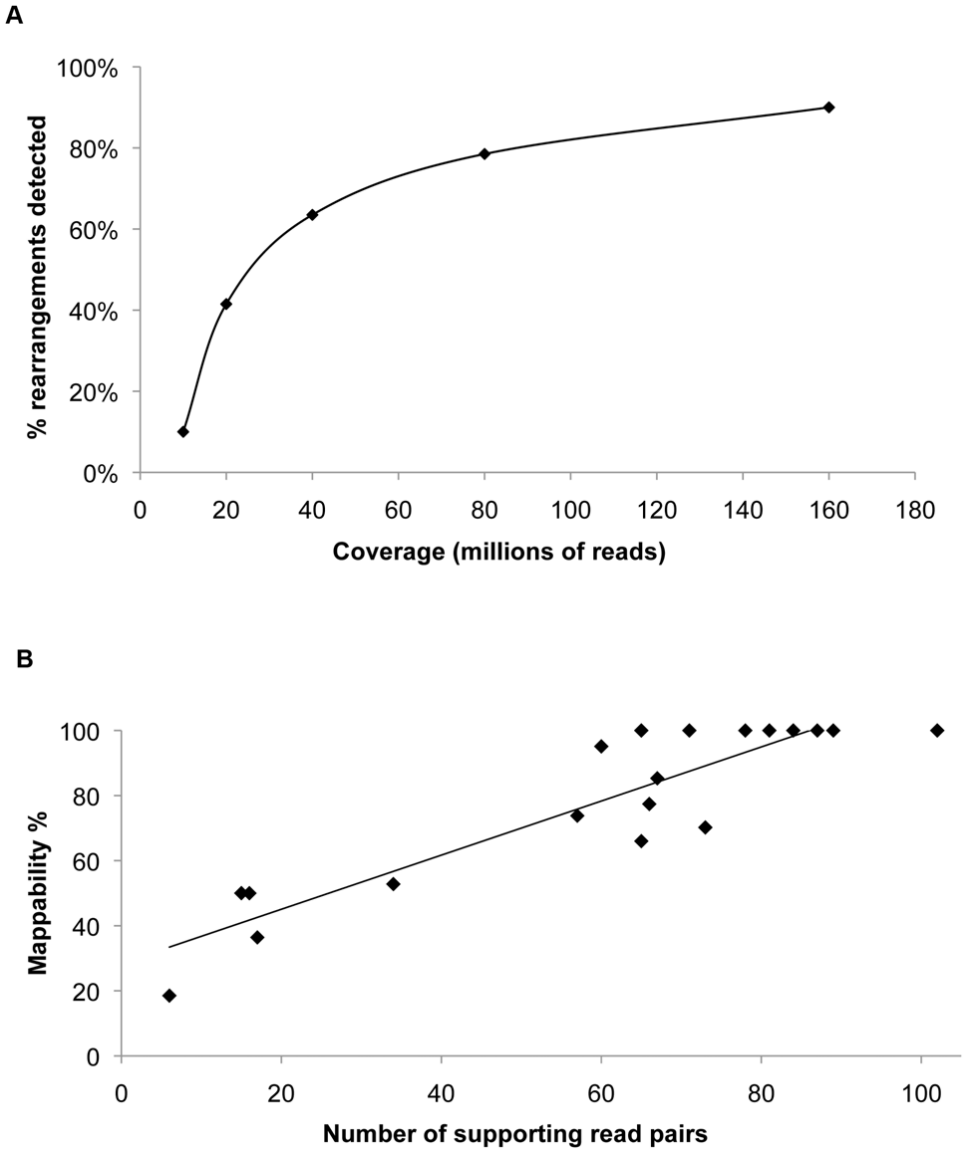
Paired-end sequencing simulations. A) Detection of SVs as a function of coverage, B) Number of supporting reads as a function of mappability.

PE sequencing proved to be an efficient method for SV detection at coverage levels corresponding to 80 or more million read pairs. 90% of events in our simulated rearranged genome were detected with 160 million read pairs, about the minimum currently obtainable from a single lane using the Illumina HiSeq platform (Fig. 1A). As expected, detectability of a certain rearrangement strongly depended on the breakpoint microenvironment, with more coverage needed to detect events in regions of lower mappability (Fig. 1B). When assessing false positives, we found that 97% of total SV calls were attributed to reads with more than one equally valid mapping position. These reads originate from various repetitive genomic regions (such as centromeric satellite sequences, retroelements, RNA genes, etc.) and had to be removed from the analysis. After examining BWA mapping quality scores of reads contributing to real and false positives, we chose a cutoff of 23 for our analysis (for further discussion, see “False positives arising from BWA alignment errors*”*). It should be noted that cutoff is chosen based on the desired ratio of real and false positives, with lower cutoff increasing sensitivity at the expense of specificity. After applying the BWA mapping quality cutoff to our simulated datasets, we observed no more false positives related to read mapping errors. However, we noticed size-related false positives that appeared with the increasing coverage. These false positives were small deletions originating from higher end and duplications originating from the lower end of the normal DNA library fragment size distribution. To correct for insert size related false positives, we used a size cutoff of 8 standard deviations and applied it to our analysis. This parameter should be determined for each library individually, depending on the desired sensitivity: increasing the standard deviation cutoff will lead to increasing the minimal detectable deletion and duplication size. Depending on the analysis needs, it may be beneficial using lower standard deviation cutoffs together with an assessment of the number of supporting read pairs, as SVs with a higher number of supporting read pairs can indicate a real event. However, this approach should be used with caution when analyzing tumor samples where loss or gain of copy number can lead to false conclusions.

Simulations of PE sequencing proved to be a useful tool in developing the data filtering strategy. After optimizing the initial parameters described above and removing all false positive calls from simulated datasets, SV calls in the experimental dataset could be attributed to the sample and the experimental procedure itself, rather than analysis artefacts. Simulations were also useful as a means to predict necessary coverage for detecting certain types of events. Importantly, when relating simulations to the experimental data analysis, it has to be taken into account that expected frequency of rearrangements, and hence the needed coverage, will normally be 50% due to the diploid nature of the genome. In case of heteroclonal or impure samples (the usual case when dealing with tumor samples), this frequency is expected to be even lower.

### Data Filtering

**Figure 2:**
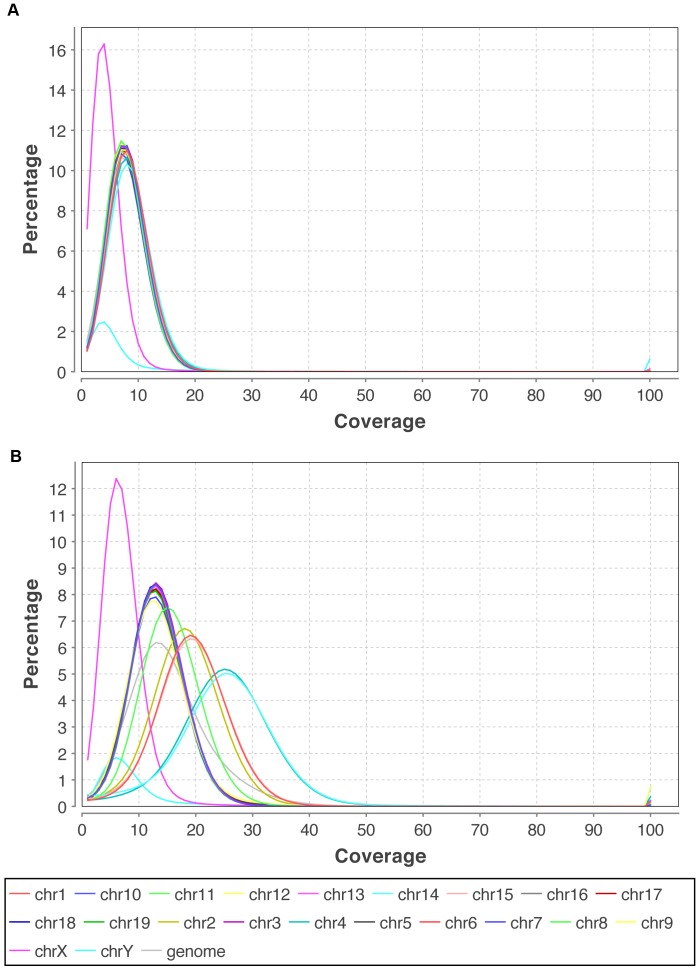
Relative coverage distribution. A) Tumor dataset, B) Control dataset. Tumor dataset shows differential relative distribution of coverage due to genomic instability. Chromosome number changes are evident for chr1, chr2, chr15 (∼3 copies), chr4 and chr14 (∼4 copies), chr8 (∼2.5 copies).

As our experimental dataset, we chose an uncharacterized thymic lymphoma obtained from a Rag2^c/c^p53^−/−^ mouse. Thymic lymphomas arising spontaneously in this mouse model harbor a large number of structural rearrangements such as translocations, large deletions and amplifications [22]. Illumina’s paired-end sequencing was chosen over the mate pair strategy, which we abandoned in the early course of this work due to difficulties in DNA library preparation. We sequenced two genomic libraries, one obtained from the solid tumor tissue and the other from the liver of the same animal (germline control). We found the control library to be essential due to a large number of germline SVs originating from remains of a 129 strain background (the mouse was initially created as a 129SvEv/C57BL6 hybrid). The tumor and control library were sequenced to 17x and 9x physical coverage, respectively (Table 2, Fig. 2).

**Table 2:** Data statistics.

	TUMOR DATASET	CONTROL DATASET
Total number of unfiltered Ilumina HiSeq read pairs	445046442	231370662
Unmapped	13350529	7275688
Duplicated read pairs/percentage	40573999/9.1%	18667266/8.1%
Mean insert size	315	299
Standard deviation	44	40
Read length	50	50
Coverage**	17x	9x
Anomalously mapped* (same chromosome)	715169	379049
Anomalously mapped* (different chromosomes)	2932221	1258825

* Number of anomalous read pairs after removing duplicated and unmapped read pairs.

** Calculated as physical coverage.

**Figure 3:**
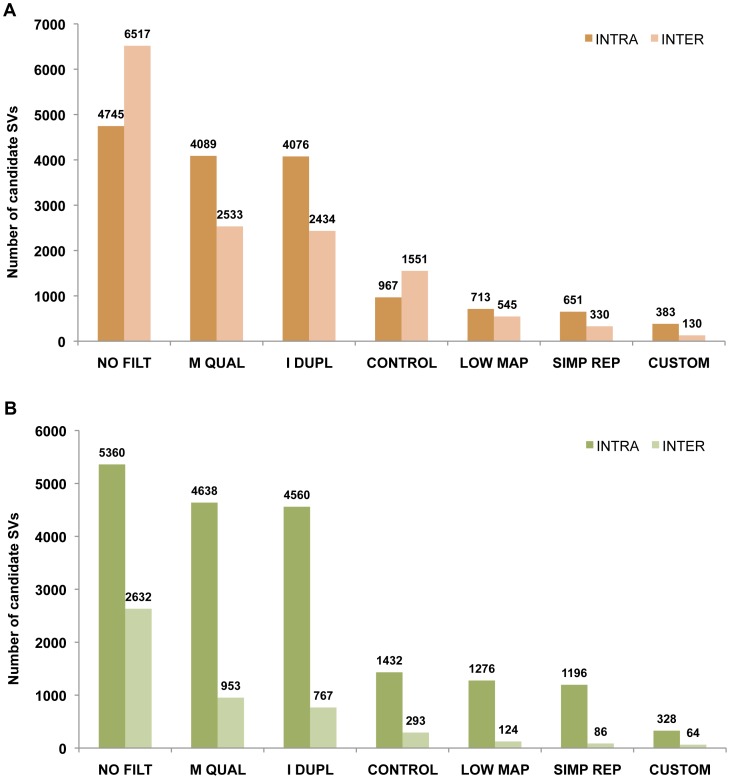
Tumor-specific SVs: data filtering. Graph shows total number of SV calls by SVDetect (A) or BreakDancer (B), as consecutive filtering steps are applied. NO FILT- No filtering (except removal of perfect PCR duplicates and reads with zero BWA mapping quality), M QUAL- Removing reads with <23 BWA mapping quality, I DUPL- Removing reads in the category of “imperfect duplicates”, CONTROL- Comparing tumor dataset to the control, LOW MAP- Post-SV detection filtering of calls overlapping low mappability regions, SIMP REP- Post-SV detection filtering of calls overlapping simple repeats, CUSTOM- Custom filtering of remaining calls based on the rearrangement type (see text for details).

We used SVDetect (Fig. 3A) and BreakDancer (Fig. 3B) to call initial SVs, as these are the two most widely used large structural variant detection programs applicable to 50 bp read PE data. Generally, the analysis using the BreakDancer initially produced more intrachromosomal and less interchromosomal SV calls compared to SVDetect, perhaps due to differences in the clustering strategy. The same analysis parameters and filtering procedure was applied to both programs, yielding similar results at the end.

In contrast to simulations, analysis of experimental data led to a large number of false positive calls after applying initially established analysis parameters described above. We define these false positives as events supported by reads mapping to repetitive genomic regions, as well as those that span regions with retroelement activity. The number of false positives was especially large among interchromosomal SVs, explained by the higher likelihood of a repetitive read being misaligned to a chromosome different from its mate. In order to find and validate real tumor-specific variants, it was necessary to analyze the source of these calls and reduce them to a manageable number. We identified 3 main types of false positive calls, depending on their source: 1) false positives related to variation between mouse strains, 2) false positives arising from alignment errors, and 3) false positives related to PCR duplicates originating from sample preparation combined with sequencing errors. We developed different pre- and post-detection filtering procedures in order to work around these challenges.

### False Positives Related to Structural Variation between Laboratory Mouse Strains

**Figure 4:**
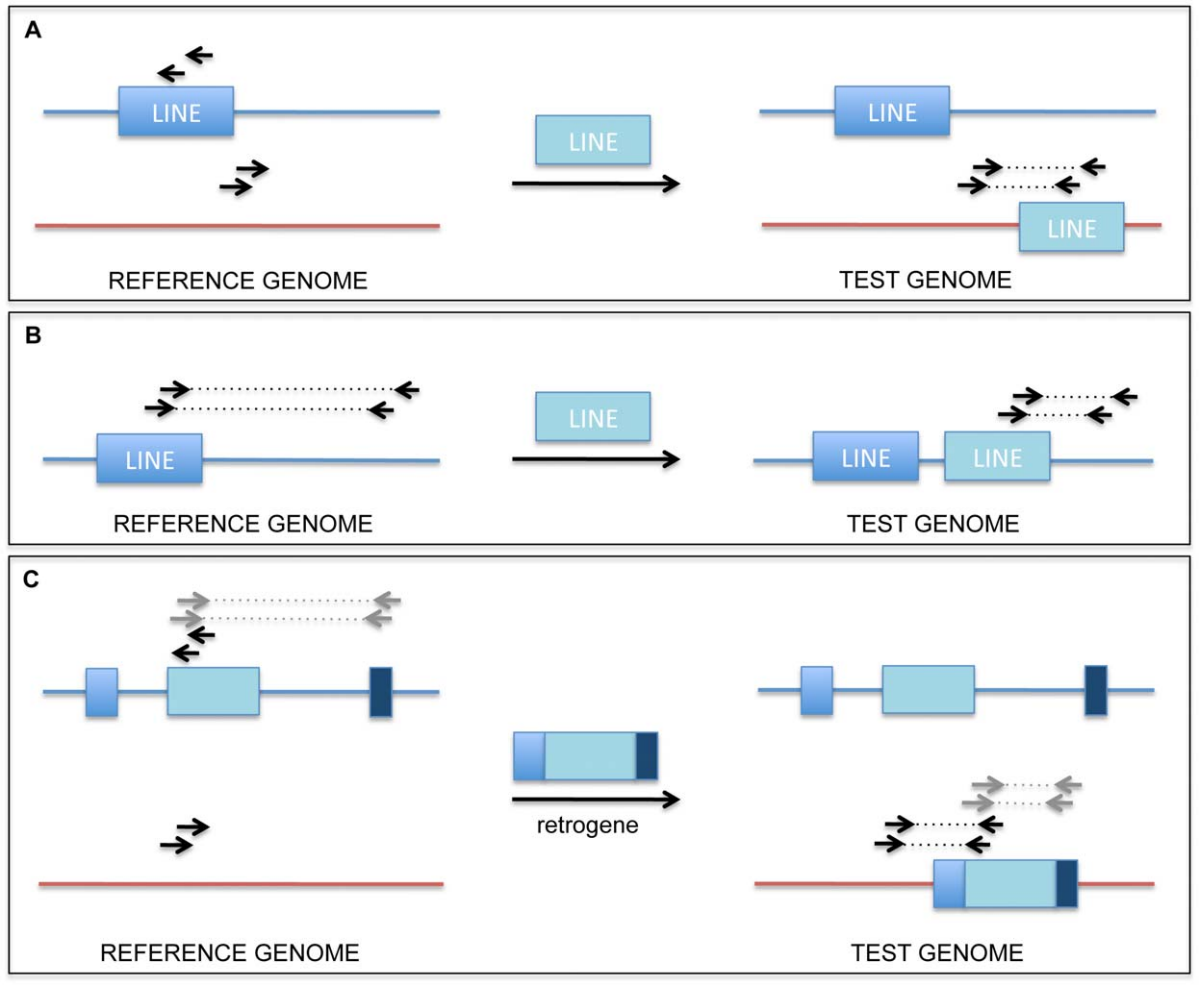
Retrotransposon and retrogene insertions leading to false positive calls. A) Retrotransposon insertion to a different chromosome leading to a false translocation call, B) Retrotransposon insertion to the same chromosome as the original leading to a false deletion call, C) Reverse transcribed intronless gene (retrogene) insertion to a different chromosome leading to false translocation and deletion calls.

Structural variation among commonly used laboratory mouse strains, similar to structural variation between individual humans, has already been documented in great detail [23], [24], [25]. Most knock-in mice, including the one used in this study, can be classified as hybrid strains, even if the animals were backcrossed a number of times to the reference genome strain (C57BL/6J). Observed SVs can mostly be attributed to germline retroelement activity, and are manifested as insertions of SINE, LINE and LTR elements as well as reverse-transcribed intronless genes (retrogenes). When an experimental dataset is compared to the C57BL/6J reference genome, several types of structural variants are called. Most commonly, retroelement insertions present in the reference, but missing in the sample strain, will be called as deletions, while those present in the sample strain, but missing in the reference, will be called as balanced translocations. Insertions of retrogenes can be recognized as a number of deletions encompassing introns, accompanied by a translocation call from the chromosome of origin to the recipient chromosome (Fig. 4).

In order to filter out germline SVs described above, we found it necessary to obtain a control dataset by sequencing normal tissue originating from the same animal. In this study, a control dataset was prepared using liver tissue and compared to the tumor dataset. Using this strategy, we were able to remove most germline SVs. However, certain SVs failed to be detected as germline, due to lack of overlap between supporting read pairs. Therefore, we found it necessary to examine each SV manually for potentially missed overlap with the control. Even after applying the comparison procedure, a number of events we identified as high quality candidates were validated as germline (30% of intrachromosomal and 50% of interchromosomal SVs). This result can be attributed to lower coverage in our control dataset, leading to lower sensitivity of germline SV detection. Aneuploidy of tumor tissue (additional copies of some chromosomes or loss of others) creates local differences in coverage between the tumor and control dataset, which adds to the complexity of the analysis (Fig. 2).

### False Positives Arising from BWA Alignment Errors

**Figure 5:**
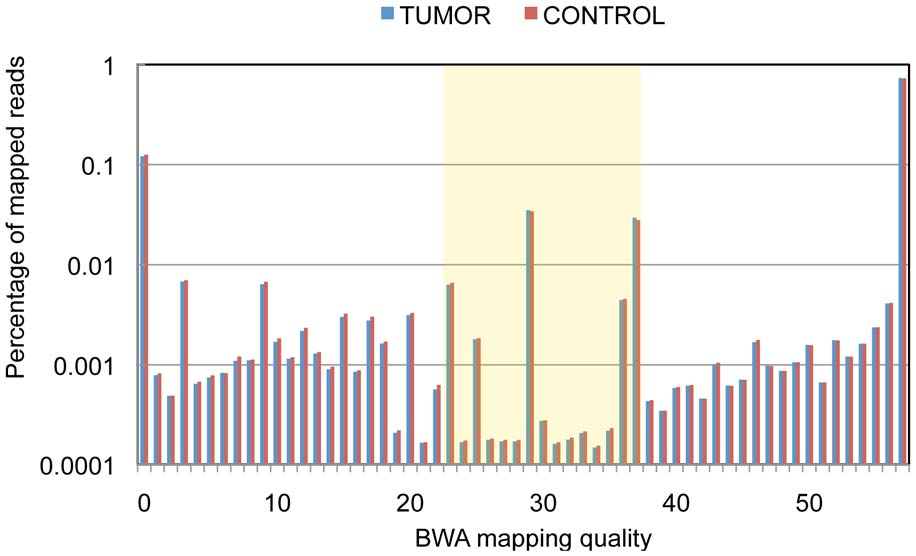
Mapping quality distribution. Discordant reads with mapping qualities above 22 are used for this analysis (box).

To remove false positives related to alignment errors, we tested the effect of BWA mapping quality score-based filtering on the number of resulting SV calls. Although BWA authors designate reads with 0–10 mapping quality as “unreliably mapped” [26], we found the best cutoff range for mapping quality score in our experiment to be 0–22 (Fig. 5). To partially correct for undesired removal of real SV candidates in less unique genomic regions, calls with large numbers of supporting read pairs were examined manually. However, none of the examined removed SVs could be designated as high quality candidates, since they all involved genomic regions of low mappability. After applying this read mapping quality filter before any other filtering is applied, the number of called SVs was reduced to 85% for intrachromosomal and 36–39% for interchromosomal events (Fig. 3).

To further reduce the number of SV calls resulting from misalignment of reads originating from repetitive regions, we tested the strategy of removing SVs with overlap with the RepeatMasker [27] and the simple repeats track of the UCSC Genome Browser. We found that RepeatMasker strategy reduces the number of false positive calls significantly, but filters out 12% of previously validated rearrangements, including some with potential biological importance (eg. Pten deletion). Importantly, reads coming from RepeatMasker annotated regions are not necessarily difficult to map uniquely, since this track contains many ancient repeated elements that have significantly diverged through evolution. RepeatMasker filtering strategy was finally used only to identify high confidence candidates among interchromosomal events with low numbers of supporting read pairs. In contrast to the RepeatMasker, overlap with simple repeats track was found to be successful in filtering out alignment error related false positives only.

As another strategy of dealing with repetitive element-related false positives, we tested the efficiency of filtering SVs against the low mappability regions, calculated based on the mappability data of the UCSC Genome Browser (see Materials and Methods). This strategy proved to very successful, removing significant numbers of false positive calls, especially efficient in the case of interchromosomal SVs (Fig. 3).

### False Positives Related to Errors in Duplicate Calling

**Figure 6:**
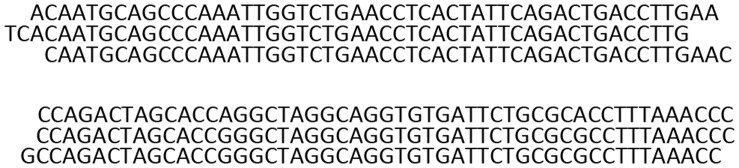
An example of imperfect duplicates. Three read pairs, likely originating from one DNA fragment, showing 1–2 bp offset in genomic coordinates.

In the course of our analysis, we observed false positives called from small clusters of 2 or 3 read pairs, with both reads mapping at positions 0–2 bp away from one another (Fig. 6). As already discussed by others in the field [28], most of these “imperfect duplicates” probably originated from one DNA fragment and diverged either during PCR amplification, perhaps due to template strand slipping, or sequencing errors at the beginning or the end of the read during the sequencing procedure. These bona fide duplicates cannot be removed using existing tools such as Picard’s MarkDuplicates since they do not have identical mapping positions. Percentage of imperfect duplicates appears to be correlated with the percentage of perfect PCR duplicates: specific datasets with high perfect duplicate percentage will show higher percentage of imperfect duplicates (M. Mijušković, results not part of this study).

We defined imperfect duplicates as pairs with the same mapping position of both reads with the possible offset up to 2 bp. Detection of these duplicates was done during clustering of discordant read pairs by SVDetect or BreakDancer, using different strategies (see Materials and Methods). After applying this filter, the number of intrachromosomal and interchromosomal SVs was reduced by 0.3–1.7% and 3.9–19.5%, respectively (Figure 3). Importantly, these numbers might underestimate the total imperfect duplicate percentage since in this case they were detected after removing low mapping quality reads.

### Validating Structural Variants

We created the final list of 61 high confidence SVs (see Materials and Methods) after manual examination of 381 intrachromosomal and 130 interchromosomal SVs detected by SVDetect and 328 intrachromosomal and 64 interchromosomal SVs detected by BreakDancer obtained after applying our filtering procedure. The majority of these calls, called by both programs, were found to either be a result of alignment errors related to repeats (59%), or previously unidentified germline SVs such as retroelement or retrogene insertions (23%). BreakDancer detected only a subset of high confidence SVs found by SVDetect (47 out of 61), even before any filtering was applied, perhaps due to differences in the clustering algorithm.

We used PCR to test 57 intrachromosomal and 4 interchromosomal high confidence SVs found by the BreakDancer and/or SVDetect (Table S1). From this set, we validated 23 large (1–539 kb) deletions, 10 inversions, 5 duplications and 2 translocations as tumor-specific, and the specificity of the PCR products was confirmed by Sanger sequencing (Table 3). Thus, 40 of the 61 high confidence SVs identified by our method were validated as tumor specific SVs. The other 19 intrachromosomal and 2 interchromosomal events were PCR validated as germline SVs. 16 out of 21 of these SVs had at least one supporting read pair in the original control dataset and failed to be detected due to our 2 supporting read cutoff. These false positives can be avoided either by sequencing the control dataset to higher coverage, when possible, or examining the control dataset using the 1 read pair cutoff.

**Table 3:** Tumor-specific SVs validated by PCR and Sanger sequencing.

Type	Location	Size (kb)	Read pairs (#)	Gene(s)
TRANSLOCATION	chr14-X	–	21	TCR alpha
	chrX-14	–	14	TCR alpha
DELETION	Chr3	59	3	Pag1
	Chr5	32.1	3	Agpat9
	Chr6	261.8	8	TCR beta
	Chr6	479.7	5	TCR beta
	Chr6	471.4	3	TCR beta
	Chr6	145.1	5	Ig kappa
	Chr12	365.7	19	Tmem179
	Chr12	53.6	29	–
	Chr12	456.8	6	Aspg, Tmem179, Mir3073, Kif26a
	Chr12	56.1	5	Inf2, Adssl1, Siva1
	Chr12	14.3	2	Akt1
	Chr12	1.3	19	IgH
	Chr12	54	5	Tdrd9
	Chr12	10.9	32	–
	Chr12	104.4	2	Adssl1, Siva1, Akt1, Zbtb42
	Chr13	25.6	29	TCR gamma
	Chr14	154	10	TCR alpha
	Chr14	366	5	TCR alpha
	Chr14	481.9	2	TCR alpha
	Chr16	23.1	4	Runx1
	Chr18	270.7	5	Arhgap26, Nr3c1
	Chr19	538.9	12	Pten
	ChrX	1.1	17	–
INVERSION	Chr1	106.1	3	–
	Chr1	1.2	11	–
	Chr12	164.2	5	Inf2, Adssl1, Siva1, Akt1, Zbtb42, AW555464
	Chr12	3.7	5	Inf2
	Chr12	6	3	Adssl1
	Chr12	5.1	28	Tmem179
	Chr12	121.4	8	Tdrd9, Aspg
	Chr12	533	7	Tdrd9, Aspg, Mir203, Mir3073, Kif26a, Tmem179
	Chr12	1.2	7	Inf2
	Chr14	1	8	TCR alpha
DUPLICATION	Chr8	495.9	3	Cdh13
	Chr12	28.9	16	Tdrd9
	Chr12	4969	32	IgH
	Chr12	3094.8	16	>20 genes
	Chr12	3094.4	4	>20 genes

Among validated tumor-specific SVs, we found several tumor-suppressor gene deletions, as well as some expected canonical antigen receptor gene rearrangements (Table 3). Notably, two tumor-specific translocations, two inversions and one validated tumor-specific duplication show signs of a complex rearrangement [29].

### Conclusions

First, our work shows that simulating paired-end sequencing can be an effective way to develop the analysis strategy, predict coverage necessary to detect DNA breakpoints in different genomic environments and to separate sources of false positive calls into sample related and those that arise due to analysis artefacts.

Second, we have found that a control dataset obtained from the same animal is essential to reduce a large number of germline SVs that exist between commonly used laboratory mouse strains, even in cases when the animals are backcrossed a number of times to the reference genome strain.

Third, we have defined two types of duplicated reads leading to false SV prediction, both arising from PCR over-amplification during sample preparation: perfect duplicates, with matching genomic coordinates, and those with 1–2 bp coordinate offset that are not detected using existing tools. We present a method to remove SVs resulting from those reads using either SVDetect or BreakDancer.

Fourth, we find that removing reads with low BWA mapping quality, as well as SV calls that overlap with genomic regions of low mappability, is a very efficient way to filter our large numbers of false positives that arise due to alignment errors.

Finally, using this method, we validated a fairly large number of true tumor-specific SVs from a rather small dataset. Starting with a large number of candidate events, we were able to rapidly discard majority of false positives and focus on a tractable number of candidates for manual analysis (∼5% of the initial number of calls from this dataset). We validated our filtering method with two widely used SV detection programs, SVDetect and BreakDancer, showing that it is universally applicable, rather than being restricted to a single program and its possible shortcomings. The final number of candidate events, as well as the number of false negatives, is a function of coverage and the stringency of filtering parameters. Depending on the needs of the experiment, these parameters can be set to a desired level in order to achieve an acceptable number of false positives vs. false negatives.

Our method should be applicable for future work in model organisms as well as in human tumors. In the clinical context, higher coverage would be needed to reduce the number of undetected germline SVs, as well as to improve the detection of low frequency somatic SVs.

## Materials and Methods

### Simulating PE Sequencing Data

Simulated PE sequencing datasets were created based on a mutated mouse reference genome (mm9) containing 10 translocations and 10 large deletions introduced using the EMBOSS tools (http://emboss.sourceforge.net). Illumina format fastq files were written using our PE.pl program (http://sourceforge.net/projects/svdetection) that selects random positions in the user-provided genome, normalized for different chromosome lengths. User-defined parameters include the number of read pairs, read length, mean insert size and standard deviation.

### Obtaining Experimental Data

Thymoma and liver (control) tissue were harvested from a Rag2^c/c^p53^−/−^ mouse [22], a 129SvEv/C57BL6 hybrid strain, and genomic DNA was purified using Blood & Cell Culture DNA Maxi Kit (Qiagen, #13362). Paired-end libraries were generated from 1 ug starting genomic material from both tissues using TruSeq DNA v2 Sample Prep Kit (Illumina, #FC-121-2001) according to manufacturer’s recommendations. Optimal PCR amplification of adapter-ligated DNA was determined using a FlashGel DNA System (Lonza, #57026). Libraries were analyzed for size distribution using Agilent 2100 Bioanalyzer (Agilent Technologies, #5067-4626) and the DNA concentration was determined using Qubit dsDNA HS Assay Kit (Life Technologies, #Q32851). Samples were sequenced on Illumina HiSeq 2000 using TruSeq PE Cluster Kit v3 (Illumina, #PE-401-3001) and TruSeq SBS Kit v3 (Illumina, #FC-401-3002), according to manufacturer’s recommendations. Two lanes were used to sequence the tumor and one lane for the control DNA library (SRA accession number: SRA055958).

### PE Read Alignment and Quality Filtering

Fastq files were generated using Casava 1.8 (Illumina) and reads were aligned using BWA [26]. Output files were manipulated by Samtools as needed [30]. Perfect PCR duplicates were removed using Picard’s MarkDuplicates tool (http://sourceforge.net/apps/mediawiki/picard). BWA-designated concordant read pairs and read pairs with low BWA mapping quality scores were removed using our own software (http://sourceforge.net/projects/svdetection), as needed.

### Calling Structural Variants and Removing Imperfect Duplicates

SVDetect [20] or BreakDancer [21] were used to call intrachromosomal and interchromosomal rearrangements from discordant, quality pre-filtered read pairs. Mean insert size and standard deviation used in this analysis were obtained by Picard’s InsertSizeMetrics tool (http://sourceforge.net/apps/mediawiki/picard). SVDetect and BreakDancer were configured to detect rearrangements with 2 or more supporting read pairs using 8 times standard deviation as threshold for both deletions and duplications. SVDetect built-in “compare” function was used for comparison of the tumor and control datasets. When comparing the calls, the option for comparing only the same SV type was turned off. For SV detection with BreakDancer, tumor to normal comparison was done using BEDTools [31].

To remove PCR duplicates with 1–2 bp offset in coordinates (“imperfect duplicates”), we manipulated the output file created by the SVDetect "linking" function using our own software (http://sourceforge.net/projects/svdetection). This file lists clusters of read pairs supporting the same rearrangement and contains coordinates of individual supporting reads. Pairs where both reads are positioned 0, 1 or 2 base pairs away from each other, in the same orientation, were removed as imperfect duplicates. In BreakDancer-based SV analysis, we changed the minimum SV anchoring region setting to 3, in order to avoid SVs being called from clusters of imperfect PCR duplicates. We also examined reads supporting SV calls in BreakDancer-produced bed files and used our own software to remove any SVs resulting from imperfect duplicates (http://sourceforge.net/projects/svdetection).

### Defining High Confidence SV Candidates

Structural variants called by SVDetect were additionally filtered based on the overlap with low mappability regions, simple repeats and RepeatMasker data extracted from the UCSC Table Browser [32]. Overlap between these regions and SVDetect links was assessed using Galaxy tools [33], [34], [35]. Low mappability regions were assembled as adjacent intervals of 50 bp with Duke ENCODE uniqueness scores less than 0.5 (the 50 bp sequence occurs more than 2 times in the genome). SVs with links overlapping these regions were removed, with the cutoff at 85% and 50% overlap for intrachromosomal and interchromosomal events, respectively. For overlap with simple repeat regions, the cutoff was 50% or greater. RepeatMasker overlap was used as a filter only for interchromosomal events supported by 2 or 3 read pairs, with the cutoff set to 80%. For intrachromosomal events, the additional custom filtering was applied to remove SVs called from read pairs arising from DNA fragments deviating from the expected library insert size range that were not removed by our standard deviation cutoff. To account for this, deletion size cutoff was set to 600 bp and duplication to 300 bp.

Tumor-specific SVs called by SVDetect and BreakDancer were finally examined manually to generate the list of high confidence candidates. SVs originating from alignment errors (related to repetitive genomic regions), failed tumor-control comparison filtering, as well as germline SVs (retroelement and retrogene insertions) were removed from the list or designated as low confidence candidates.

### Validation of SV Calls

High confidence SV candidates were validated by PCR using custom designed primers mapping to SV “linking” regions, in the appropriate orientation. SVs validated as tumor-specific were cloned using the TOPO® TA Cloning Kit (Invitrogen, K4500–01). For each SV, two independent clones were sequenced by the Sanger method. Resulting sequences were mapped using BLAT [36].

### Ethics Statement

This study was carried out in strict accordance with the recommendations in the Guide for the Care and Use of Laboratory Animals of the National Institutes of Health. The protocol was approved by the Institutional Animal Care and Use Committee of the University of Pennsylvania (Permit Number: 803893).

## Supporting Information

Table S1 **High confidence SVs found by the BreakDancer and SVDetect.**
(XLS)

## References

[1] Konopka JB, Watanabe SM, Singer JW, Collins SJ, Witte ON (1985) Cell lines and clinical isolates derived from Ph1-positive chronic myelogenous leukemia patients express c-abl proteins with a common structural alteration. Proc Natl Acad Sci U S A 82: 1810–4. 3856862 10.1073/pnas.82.6.1810 PMC397362

[2] Tsujimoto Y, Gorham J, Cossman J, Jaffe E, Croce CM (1985) The t(14;18) chromosome translocations involved in B-cell neoplasms result from mistakes in VDJ joining. Science 229: 1390–3. 3929382 10.1126/science.3929382

[3] Strong LC, Riccardi VM, Ferrell RE, Sparkes RS (1981) Familial retinoblastoma and chromosome 13 deletion transmitted via an insertional translocation. Science 213: 1501–3. 7280668 10.1126/science.7280668

[4] Albertson DG, Collins C, McCormick F, Gray JW (2003) Chromosome aberrations in solid tumors. Nat Genet 34: 369–76. 12923544 10.1038/ng1215

[5] Erikson J, Nishikura K, ar-Rushdi A, Finan J, Emanuel B, et al (1983) Translocation of an immunoglobulin kappa locus to a region 3′ of an unrearranged c-myc oncogene enhances c-myc transcription. Proc Natl Acad Sci U S A 80: 7581–5. 6424112 10.1073/pnas.80.24.7581 PMC534384

[6] ar-Rushdi A, Nishikura K, Erikson J, Watt R, Rovera G, et al (1983) Differential expression of the translocated and the untranslocated c-myc oncogene in Burkitt lymphoma. Science 222: 390–3. 6414084 10.1126/science.6414084

[7] Druker BJ, Talpaz M, Resta DJ, Peng B, Buchdunger E, et al (2001) Efficacy and safety of a specific inhibitor of the BCR-ABL tyrosine kinase in chronic myeloid leukemia. N Engl J Med 344: 1031–7. 11287972 10.1056/NEJM200104053441401

[8] Druker BJ, Sawyers CL, Kantarjian H, Resta DJ, Reese SF, et al (2001) Activity of a specific inhibitor of the BCR-ABL tyrosine kinase in the blast crisis of chronic myeloid leukemia and acute lymphoblastic leukemia with the Philadelphia chromosome. N Engl J Med 344: 1038–42. 11287973 10.1056/NEJM200104053441402

[9] Bentley DR, Balasubramanian S, Swerdlow HP, Smith GP, Milton J, et al (2008) Accurate whole human genome sequencing using reversible terminator chemistry. Nature 456: 53–9. 18987734 10.1038/nature07517 PMC2581791

[10] Mardis ER (2008) Next-generation DNA sequencing methods. Annu Rev Genomics Hum Genet 9: 387–402. 18576944 10.1146/annurev.genom.9.081307.164359

[11] Nowell PC (1976) The clonal evolution of tumor cell populations. Science: 23–8. 10.1126/science.959840 959840

[12] González-García I, Solé RV, Costa J (2002) Metapopulation dynamics and spatial heterogeneity in cancer. Proc Natl Acad Sci U S A 99: 13085–9. 12351679 10.1073/pnas.202139299 PMC130590

[13] Merlo LM, Pepper JW, Reid BJ, Maley CC (2006) Cancer as an evolutionary and ecological process. Nat Rev Cancer 6: 924–35. 17109012 10.1038/nrc2013

[14] Britten RJ, Kohne DE (1968) Repeated sequences in DNA. Hundreds of thousands of copies of DNA sequences have been incorporated into the genomes of higher organisms. Science 161: 529–40. 4874239 10.1126/science.161.3841.529

[15] Schmid CW, Deininger PL (1975) Sequence organization of the human genome. Cell 6: 345–58. 1052772 10.1016/0092-8674(75)90184-1

[16] Onishi-Seebacher M, Korbel JO (2011) Challenges in studying genomic structural variant formation mechanisms: the short-read dilemma and beyond. Bioessays 33: 840–50. 21959584 10.1002/bies.201100075

[17] Treangen TJ, Salzberg SL (2011) Repetitive DNA and next-generation sequencing: computational challenges and solutions. Nat Rev Genet 13: 36–46. 22124482 10.1038/nrg3117 PMC3324860

[18] Akagi K, Li J, Stephens RM, Volfovsky N, Symer DE (2008) Extensive variation between inbred mouse strains due to endogenous L1 retrotransposition. Genome Res 18: 869–80. 18381897 10.1101/gr.075770.107 PMC2413154

[19] Bennett EA, Coleman LE, Tsui C, Pittard WS, Devine SE (2004) Natural genetic variation caused by transposable elements in humans. Genetics 168: 933–51. 15514065 10.1534/genetics.104.031757 PMC1448813

[20] Zeitouni B, Boeva V, Janoueix-Lerosey I, Loeillet S, Legoix-né P, et al (2010) SVDetect: a tool to identify genomic structural variations from paired-end and mate-pair sequencing data. Bioinformatics 26: 1895–6. 20639544 10.1093/bioinformatics/btq293 PMC2905550

[21] Chen K, Wallis JW, McLellan MD, Larson DE, Kalicki JM, et al (2009) BreakDancer: an algorithm for high-resolution mapping of genomic structural variation. Nat Methods 6: 677–81. 19668202 10.1038/nmeth.1363 PMC3661775

[22] Deriano L, Chaumeil J, Coussens M, Multani A, Chou Y, et al (2011) The RAG2 C terminus suppresses genomic instability and lymphomagenesis. Nature 471: 119–23. 21368836 10.1038/nature09755 PMC3174233

[23] Quinlan AR, Clark RA, Sokolova S, Leibowitz ML, Zhang Y, et al (2010) Genome-wide mapping and assembly of structural variant breakpoints in the mouse genome. Genome Res 20: 623–35. 20308636 10.1101/gr.102970.109 PMC2860164

[24] Mills RE, Luttig CT, Larkins CE, Beauchamp A, Tsui C, et al (2006) An initial map of insertion and deletion (INDEL) variation in the human genome. Genome Res 16: 1182–90. 16902084 10.1101/gr.4565806 PMC1557762

[25] Korbel JO, Urban AE, Affourtit JP, Godwin B, Grubert F, et al (2007) Paired-end mapping reveals extensive structural variation in the human genome. Science 318: 420–6. 17901297 10.1126/science.1149504 PMC2674581

[26] Li H, Durbin R (2009) Fast and accurate short read alignment with Burrows-Wheeler transform. Bioinformatics 25: 1754–1760. 19451168 10.1093/bioinformatics/btp324 PMC2705234

[27] Smit AFA, Hubley R, Green P. RepeatMasker Open-3.0. Available: http://www.repeatmasker.org. 1996–2010. Accessed 2012 Oct 2.

[28] Hall IM, Quinlan AR (2012) Detection and interpretation of genomic structural variation in mammals. Methods Mol Biol 838: 225–48. 22228015 10.1007/978-1-61779-507-7_11

[29] Quinlan AR, Hall IM (2012) Characterizing complex structural variation in germline and somatic genomes. Trends Genet 28: 43–53. 22094265 10.1016/j.tig.2011.10.002 PMC3249479

[30] Li H*, Handsaker B*, Wysoker A, Fennell T, Ruan J, et al (2009) The Sequence alignment/map (SAM) format and SAMtools. Bioinformatics 25: 2078–9. 19505943 10.1093/bioinformatics/btp352 PMC2723002

[31] Quinlan AR, Hall IM (2010) BEDTools: a flexible suite of utilities for comparing genomic features. Bioinformatics 26 6: 841–842. 10.1093/bioinformatics/btq033 PMC2832824 20110278

[32] Karolchik D, Hinrichs AS, Furey TS, Roskin KM, Sugnet CW, et al (2004) The UCSC Table Browser data retrieval tool. Nucleic Acids Res 32: D493–6. 14681465 10.1093/nar/gkh103 PMC308837

[33] Blankenberg D, Von Kuster G, Coraor N, Ananda G, Lazarus R, et al.. (2010) Galaxy: a web-based genome analysis tool for experimentalists. Current Protocols in Molecular Biology. Chapter 19: Unit 19.10.1–21. 10.1002/0471142727.mb1910s89 PMC4264107 20069535

[34] Giardine B, Riemer C, Hardison RC, Burhans R, Elnitski L, et al (2005) Galaxy: a platform for interactive large-scale genome analysis. Genome Research 15: 1451–5. 16169926 10.1101/gr.4086505 PMC1240089

[35] Goecks J, Nekrutenko A (2010) Taylor J and The Galaxy Team (2010) Galaxy: a comprehensive approach for supporting accessible, reproducible, and transparent computational research in the life sciences. Genome Biol 11: R86. 20738864 10.1186/gb-2010-11-8-r86 PMC2945788

[36] Kent WJ (2002) BLAT - the BLAST-like alignment tool. Genome Res 12: 656–64. 11932250 10.1101/gr.229202 PMC187518

